# Modeling Hall–Petch Relationships of Alloyed Nanostructured Materials Regarding Bonding Nature at Grain Boundaries

**DOI:** 10.3390/ma19040767

**Published:** 2026-02-15

**Authors:** Haibo Lv, Feng Jiang, Yongfu Zhu

**Affiliations:** 1 College of Mechanical and Electrical Engineering, Jilin University of Chemical Technology, Jilin 132022, China; lhbjlict@163.com (H.L.); jf19840830@sina.com (F.J.); 2 Key Laboratory of Automobile Materials, Ministry of Education and School of Materials Science and Engineering, Jilin University, Changchun 130022, China

**Keywords:** Hall–Petch relationship, nanostructured materials, alloying, interface energy

## Abstract

Based on a thermodynamic approach, a unified formula was proposed to describe distinct Hall–Petch relationships (HPRs) of unalloyed nanostructured materials (u-NSs: Fe, Cu, Ni, Pd, and Mo) and alloyed ones with low- or high-melting temperature alloying metals (low-*T*_m_ or high-*T*_m_ a-NSs: Ni*_x_*Mo_1−*x*_, Fe*_x_*Zr_1−*x*_, Ni*_x_*Cu_1−*x*_, and Fe*_x_*Cu_1−*x*_). As the grain size decreases to several nanometers, the yield strength first increases and then decreases for u-NSs and low-*T*_m_ a-NSs, obeying the inverse HPR (IHPR), while it monotonically increases for high-*T*_m_ a-NSs. For the former, the decrease is induced by the reduction in activation energies of interface migration and dislocation gliding, along with the thermally driven decline, lattice expansion, and bond weakening of interface atoms. In the latter case, the monotonic increase or the elimination of IHPR is relevant to the negative interface energy induced by the segregation of alloying atoms at grain boundaries. Our predictions are validated by the available experimental results.

## 1. Introduction

Nanostructured materials (NSs) are commonly used in various fields due to their high strength/weight ratio compared to coarse-grained counterparts [1,2,3]. The mechanical properties of unalloyed NSs (u-NSs) have aroused much attention thanks to their high strength, which is linked to their small grain size (*D*) [4]. When *D* > 30 nm, for example, the yield strength (σ) increases with lowering *D*. This is the Hall–Petch relationship (HPR) [5,6], and, considering the temperature effect, it can be given as [7,8,9]

(1) $$\sigma \left(T,D\right)=\sigma \left(T,\infty \right)+k_{d}\left(T\right)D^{-1/2}$$

where *T* is the working temperature, σ(*T*,∞) is the friction stress with bulk ∞, and *k*_d_(*T*) is a constant depending on the resistance to dislocation movement at grain boundaries (GBs). As *D* decreases, the GB/volume ratio increases, resulting in enhanced dislocation pile-ups at GBs, and then increased σ(*T*,*D*) [10,11]. As *D* decreases further, however, σ(*T*,*D*) does not always increase but can begin to decrease below a critical size (*D*_c_), which is the inverse HPR (IHPR) [11]. IHPR has also been observed for alloyed NSs with low-melting-temperature (*T*_m_) alloying metals (low-*T*_m_ a-NSs) [12,13]. However, such a decrease in σ(*T*,*D*) is absent for a-NSs with high-*T*_m_ alloying metals (high-*T*_m_ a-NSs) [14,15]. To further our understanding of the strengthening mechanisms of NSs, it is necessary to elucidate distinct relationships for u-NSs and a-NSs. Several classical models have been proposed for IHPR, such as the dislocation-based model [7,16], the diffusion-based model [17], the GB-shearing model [18], and the grain-boundary phase or bonding model [19,20], but they fail to elucidate the elimination of IHPR for high-*T*_m_ a-NSs.

Based on Lindemann’s melting criterion and Mott’s expression of vibrational entropy (*S*_vib_), our group proposed that the decrease in *T*_m_(*D*) of nanocrystals (NCs) induced by surface coordination imperfection can be modeled as $T_{m}^{\mathrm{NC}}$(*D*)/*T*_m_(∞) = exp[(−2*S*_vib_/3*R*)/(*D*/*D*_0_ − 1)] [21,22], where *S*_vib_ ≈ *S*_m_ = *H*_m_/*T*_m_, with *S*_m_ being the melting entropy and *H*_m_ the melting enthalpy; *R* is the ideal gas constant; and *D*_0_ = 6*h* denotes the critical diameter, with *h* as the atomic diameter, where almost all atoms of the NC are located on the surface and the crystalline structure is no longer stable. Using such a *D*-dependent $T_{m}^{\mathrm{NC}}$(*D*) formula and a phenomenological equation with σ(*T*,*D*) relying on the conventional GB gliding through boundary diffusion [9,23], where boundary diffusion or analogous behavior is also important at room temperature for NSs [24,25,26], a thermodynamic model for σ(*T*,*D*) of NSs at room temperature and above was proposed as [27]

(2) $$\sigma \left(T,D\right)=\sigma^{\prime }\left(\infty \right)+\left[k_{t}+{k_{d}}^{\prime }\left(T\right)D^{-1/2}\right]\exp[\frac{T_{m}^{\mathrm{NC}}\left(D\right)}{2T}]$$

where σ′(∞) is a *T*-independent term, *k*_t_ is a constant, and *k*_d_′(*T*) = *k*_d_(*T*)/exp[$T_{m}^{\mathrm{NC}}$(*D*)/(2*T*)]. The σ′(∞), *k*_t_, and *k*_d_′(*T*) values can be figured out by fitting results from the literature. However, since the grains of NSs are different to those of NCs with free surfaces, the aforesaid $T_{m}^{\mathrm{NC}}$(*D*) formula in Equation (2) is inappropriate for plotting the HPR or IHPR curves of NSs. Specifically, the grains of NSs possess weaker bond deficits with a lower interface energy compared to free NCs, resulting in the variation in thermal properties of NSs differing to that of free NCs [28,29]. Moreover, Equation (2) also fails to elucidate the elimination of IHPR for high-*T*_m_ a-NSs. A comprehensive model effective to elucidate distinct relationships between *D* and σ(*T*,*D*) of u-NSs and a-NSs is still required.

Attempts have been made to study the Young’s modulus (*Y*) of NSs, and it was found that the *Y*(*T*,*D*) of NSs decreases on lowering *D*, and that such a decrease is smaller than that of free NCs [30,31]. Using in situ tensile tests, moreover, Zhu et al. [32] found that the *Y*(*T*,*D*), σ(*T*,*D*), and ultimate tensile strength of Ag nanowires increase on lowering *D*, and proposed that the *D*-dependence of σ(*T*,*D*) is mainly caused by the stiffening size effect of *Y*(*T*,*D*). One may thus expect that the σ(*T*,*D*) of NSs can be worked out by solving the *D*-dependent *Y*(*T*,*D*). By considering the effects of thermally driven effect, lattice expansion, and bond weakening concerning the coordination imperfection of GBs, a model regarding the *D*-dependent *Y*(*T*,*D*) of NSs was proposed as [29]

(3) $$\frac{Y\left(T,D\right)}{Y\left(T,\infty \right)}=\frac{Y_{T}\left(T,D\right)}{Y\left(T,\infty \right)}\frac{Y_{B}\left(0,D\right)}{Y\left(0,\infty \right)}$$

where *Y*_T_(*T*,*D*)/*Y*(*T*,∞) denotes the thermally driven effect associated with $T_{m}^{\mathrm{NS}}$(*D*), and *Y*_B_(0,*D*)/*Y*(0,∞) denotes the effects of lattice expansion and bond weakening. Equation (3) indicates the importance of the thermally driven effect, lattice expansion and bond weakening in σ(*T*,*D*) variation in NSs. In addition, since the local energy density decreases as the bond energy declines, σ(*T*,*D*) should also decrease according to the proportional relationship between σ(*T*,*D*) and energy density [33]. Furthermore, in a-NSs, alloying atoms tend to aggregate at GBs, where they form new bonds and thereby alter the nature of the GBs [14,34]. Specifically, when these newly formed bonds are weaker than the interior bonds of the base metal (e.g., in low-*T*_m_ a-NSs), the GB energy (γ_gb_) is positive, indicating that additional energy is required to form such GBs. In contrast, when GB bonds are stronger than interior bonds (e.g., in high-*T*_m_ a-NSs), γ_gb_ becomes negative, implying that the GBs are energetically more stable than the bulk of the base metals. This fundamental change in GB energetics leads to distinct variation trends for σ(*T*,*D*). However, these contributions have not previously been considered in evaluating the σ(*T*,*D*) of NSs. It is notable that this linkage between the *T*_m_ of alloying elements and the sign of γ_gb_ should be regarded as a first-order approximation. In real NSs, γ_gb_ is also strongly influenced by segregation chemistry, complexion transitions, and interfacial phases. Incorporating such effects would be a valuable direction for future refinement of the framework.

In this contribution, we developed a unified formula to predict the σ(*T*,*D*) values of u-NSs and a-NSs by considering the interface energy based on presently available theories of fine grain strengthening, solid solution strengthening, and alloying strengthening. The roles of γ_gb_, lattice expansion, and bond weakening were also evaluated by considering the alloying effect. The predictions are in good agreement with the experimental results.

## 2. Methods

Referencing Equations (1)–(3) and combining the aforesaid proportional relationship between σ(*T*,*D*) and *Y*(*T*,*D*), σ(*T*,*D*) of NSs can be evaluated by

(4) $$\frac{\sigma \left(T,D\right)}{\sigma \left(T,\infty \right)}=1+k\left(T\right)D^{-1/2}\exp[\frac{2T_{m}^{\mathrm{NS}}\left(D\right)}{T}]\frac{\sigma_{T}\left(T,D\right)}{\sigma \left(T,\infty \right)}\frac{\sigma_{B}\left(0,D\right)}{\sigma \left(0,\infty \right)}$$

where *k*(*T*) is a constant; σ_T_(*T*,*D*)/σ(*T*,∞) = [$T_{m}^{\mathrm{NS}}$(*D*) − *T*]/[*T*_m_(∞) − *T*], denoting the thermally driven effect; and σ_B_(0,*D*)/σ(0,∞) = [*h*(∞)^3^ε(*D*)]/[*h*(*D*)^3^ε(∞)] denoting the effects of lattice expansion and bond weakening, with *h* being the lattice constant and ε the bond energy. The *k*(*T*)*D*^−1/2^exp [2$T_{m}^{\mathrm{NS}}$(*D*)/*T*] term covers the contributions from the activation energies of interface migration and dislocation gliding [20,27].

The $T_{m}^{\mathrm{NS}}$(*D*), *h*(*D*), and ε(*D*) values should be known to obtain σ(*T*,*D*) using Equation (4). Based on the aforementioned $T_{m}^{\mathrm{NC}}$(*D*) equation and considering the role of γ_gb_, $T_{m}^{\mathrm{NS}}$(*D*) was explored with [28]

(5) $$T_{m}^{\mathrm{NS}}\left(D\right)/T_{m}\left(\infty \right)=\exp\left[-\delta \left(2S_{\mathrm{vib}}/3R\right)/\left(D/D_{0}-1\right)\right]$$

where δ is the additional term induced by the difference between the free surface and the solid–solid interface, which can be given as

(6) $$\delta =1/\left[1+\left(\gamma_{\mathrm{sv}}/\gamma_{\mathrm{gb}}-1\right)\left(2S_{\mathrm{vib}}/3R+1\right)\right]$$

where γ_sv_ is the surface energy. Meanwhile, *h*(*D*) and ε(*D*) can be worked out using a hard-sphere approach and the *D*-dependent interface energy [35], as follows:

(7) h(D)/h(∞)=1+h(∞)/3D

(8) $$\varepsilon \left(D\right)/\varepsilon \left(\infty \right)=\left[E_{c}\left(D\right)/E_{c}\left(\infty \right)\right]/\left[6\left(Z_{\mathrm{gb}}/Z_{b}-1\right)h(\infty )/D+1\right]$$

where

(9) $$E_{c}\left(D\right)/E_{c}\left(\infty \right)=\left[1-1/\left(12D/D_{0}-1\right)\right]\exp\left[-\delta \left(2S_{b}/3R\right)/\left(12D/D_{0}-1\right)\right]$$

Here, *E*_c_(*D*) is the cohesive energy of NSs, and *Z*_b_ and *Z*_gb_ denote the atomic coordination numbers of bulk and GBs, respectively. *Z*_b_ = 8 and *Z*_gb_ = 7 for body-centered cubic (BCC) crystals, and *Z*_b_ = 12 and *Z*_gb_ = 11 for face-centered cubic (FCC) and hexagonal close-packed (HCP) crystals. *S*_b_ = *H*_b_/*T*_b_ is the bulk solid–vapor transition entropy, with *H*_b_ being the heat of vaporization and *T*_b_ the boiling point.

In addition, *Y*(*T*,*D*) declines as the difference between *T* and $T_{m}^{\mathrm{NS}}$(*D*) decreases [36]. On lowering *D*, the velocity of atomic movement accelerates through the thermally driven effect, altering the degree of plastic deformation. It is thus necessary to consider the influence from *T* and $T_{m}^{\mathrm{NS}}$(*D*) suppression using the thermally driven term. Substituting the *D*-dependent $T_{m}^{\mathrm{NS}}$(*D*) in Equation (5) into Equation (4), a unified formula can be developed as

(10) $$\frac{\sigma \left(T,D\right)}{\sigma \left(T,\infty \right)}=1+k\left(T\right)D^{-1/2}\exp[\frac{2T_{m}\left(\infty \right)}{T}\exp\left(-\delta \frac{2S_{\mathrm{vib}}/3R}{D/D_{0}-1}\right)]\frac{\sigma_{T}\left(T,D\right)}{\sigma \left(T,\infty \right)}\frac{\sigma_{B}\left(0,D\right)}{\sigma \left(0,\infty \right)}$$

Here, *k*(*T*) can be obtained by fitting experimental results regarding σ(*T*,*D*) as the function of *D* at a certain *T* using the least-squares method in order to ensure consistency across a wide range of reported experiments. *k*(*T*) represents an effective parameter integrating several physically meaningful contributions, similar to the classical Hall–Petch coefficient. Hence, even with only a small number of data points, Equation (10) can be adopted to predict the σ(*T*,*D*) values of u-NSs and a-NSs at any *D* in the full size range from micrometer to nanometer, including the maximums of σ(*T*,*D*) at *D*_c_.

To work out σ(*T*,*D*) with Equation (10), the δ term should be solved with the γ_gb_ associated with Equation (6). For u-NSs, γ_gb_(∞) = 4*hH*_m_*S*_vib_/(3*V*_s_*R*) with *V*_s_ being the molar volume [37]. For a-NSs, γ_gb_(∞) = *E*_cb_(∞) − *E*_cgb_(∞) [38], where *E*_cb_(∞) and *E*_cgb_(∞) denote the cohesive energies of bulk atoms and GB atoms, respectively. Also, *E*_cb_ = *Z*_b_ε_b_/2 and *E*_cgb_ = [*Z*_gb_ε_gb_ + (*Z*_b_ − *Z*_gb_)ε_b_]/2 with *Z*_gb_ = *Z*_sv_, where *Z*_sv_ is the atomic coordination number of a surface, ε_b_ and ε_gb_ are the bond energies of the bulk and GBs, respectively. Generally, NCs consist of (111) facets with *Z*_sv_ = 9 and (100) facets with *Z*_sv_ = 8 [37]. *Z*_gb_ = (*N*_100_*Z*_100_ + *N*_111_*Z*_111_)/(*N*_100_ + *N*_111_) is thus used, where *N*_100_ and *N*_111_ are the numbers of (100) and (111) facets, respectively. NCs can be described by the Wulff construction developed by minimizing γ_sv_ with a given enclosed volume [37,39,40,41], where truncated octahedra with regular hexagonal facets are regarded as the regular one [42]. Upon this, *N*_100_/*N*_111_ = 3(*n*^2^ − 4*n* + 4)/[4(3*n*^2^ − 9*n* + 7)], and *N*_total_ = 16n^3^ − 33*n*^2^ + 24*n* − 6, where *N*_total_ is the total number of atoms and *n* is the number of atoms at the edge. As *n* or *N* increases, *N*_100_/*N*_111_ increases by up to 25%. Accordingly, the equivalent *D* of the faceted cluster can be given as *D* = *N*_total_^1/3^*h* [42]. Moreover, the case of *N*_100_/*N*_111_ = 0, with the assumption that the surface consists only of (111) facets, is also calculated for comparison. For a-NSs, γ_gb_(∞) is positive for low-*T*_m_ a-NSs since *E*_cb_(∞) > *E*_cgb_(∞), but it becomes negative for high-*T*_m_ a-NSs since *E*_cb_(∞) < *E*_cgb_(∞).

## 3. Results and Discussion

**Figure 1:**
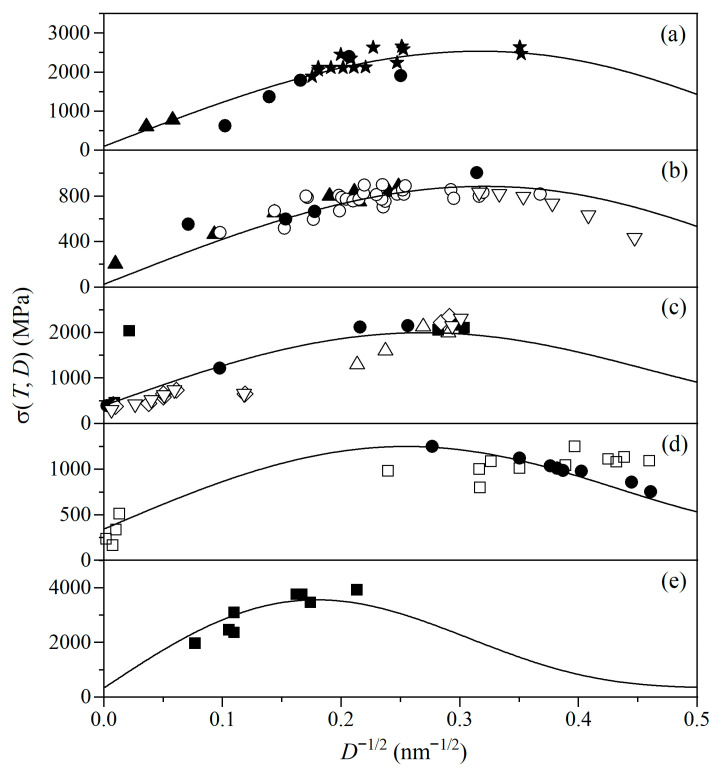
σ(*T*,*D*) as a function of *D*^−1/2^ for u-NSs of (**a**) Fe, (**b**) Cu, (**c**) Ni, (**d**) Pd, and (**e**) Mo using Equation (10). *T* = 300 K in (**a**–**d**), and *T* = 293 K in (**e**). Symbols are experimental results with ▲ [44], ★ [44], and ● [45] in (**a**) for u-NSs of Fe, ▲ [44], ▽ [25], ○ [46], and ● [45] in (**b**) for Cu u-NSs, ◇ [47], ▽ [47], ■ [48], △ [49], and ● [48] in (**c**) for u-NSs of Ni, ● [25] and □ [50] in (**d**) for u-NSs of Pd, and ■ [45] in (**e**) for u-NSs of Mo. ▽ in (**b**) is a theoretical result from ref. [51]. See Table 1 for the necessary parameters.

Figure 1 plots the σ(*T*,*D*) curves for u-NSs of Fe, Cu, Ni, Pd, and Mo. On lowering *D*, σ(*T*,*D*) increases and then reaches a maximum value when *D* = *D*_c_, where *D*_c_ is about 10–30 nm. Below *D*_c_, the further decrement of *D* leads to a shrinkage of σ(*T*,*D*). As *D* > *D*_c_, $T_{m}^{\mathrm{NS}}$(*D*) approximately equals to *T*_m_(∞). The excess volume at GBs and the interface energy are both large [43], enhancing the dislocation pile-ups and hindering the dislocation movement at GBs. This results in the increase in σ(*T*,*D*) with lowering *D*. As *D* < *D*_c_, however, $T_{m}^{\mathrm{NS}}$(*D*) decreases accompanied by interface softening [26]. As a result, the boundary diffusion or gliding begins to dominate the deformation, and σ(*T*,*D*) starts to decrease. Meanwhile, the thermally driven decline can be enhanced by the decrease in $T_{m}^{\mathrm{NS}}$(*D*), while lattice expansion and bond weakening may also enhance interface softening. All these effects lead to a reduction in σ(*T*,*D*) below *D*_c_. Notably, currently available experimental data for σ(*T*,*D*) in u-NSs and a-NSs cover only limited *D* ranges; however, the variation trends predicted by our model are broadly consistent with the experimentally observed trends within the accessible ranges.

**Table 1:** Parameters of the selected metals used in the model calculation.

	Fe	Cu	Ni	Pd	Mo
*h*(∞) (nm) [52]	0.312	0.290	0.298	0.338	0.380
*H*_m_(∞) (kJ/g-atom) [52]	13.8	13.0	17.5	17.6	32.0
*T*_m_(∞) (K) [52]	1809	1358	1726	1825	2896
*H*_b_(∞) (kJ/g-atom) [52]	347	300	378	380	600
*T*_b_(∞) (K) [52]	3134	3200	3186	3236	4926
γ_gb_(∞) (J/m^2^) ^a^	0.69	0.59	0.93	0.76	1.34
γ_sv_(∞) (J/m^2^) [53]	2.42	1.79	2.38	2.00	2.91
σ(300, ∞) (MPa)	100 [44]	25 [44]	395.5 [48]	343.3 [50]	333.3 [54]
*k*(*T*) (nm^1/2^) ^b^	6.76 × 10^−4^	1.92 × 10^−2^	2.36 × 10^−4^	8.41 × 10^−5^	2.26 × 10^−7^

^a^ *V*_s_ = 7.1, 7.1, 6.6, 8.9, and 9.4 cm^3^/g-atom for Fe, Cu, Ni, Pd, and Mo, respectively [52]. ^b^ *k*(*T*) was obtained by fitting all experimental results listed in the caption of Figure 1 with the least-squares method in terms of Equation (10). *T* = 300 K for Fe, Cu, Ni, and Pd, and *T* = 293 K for Mo.

**Figure 2:**
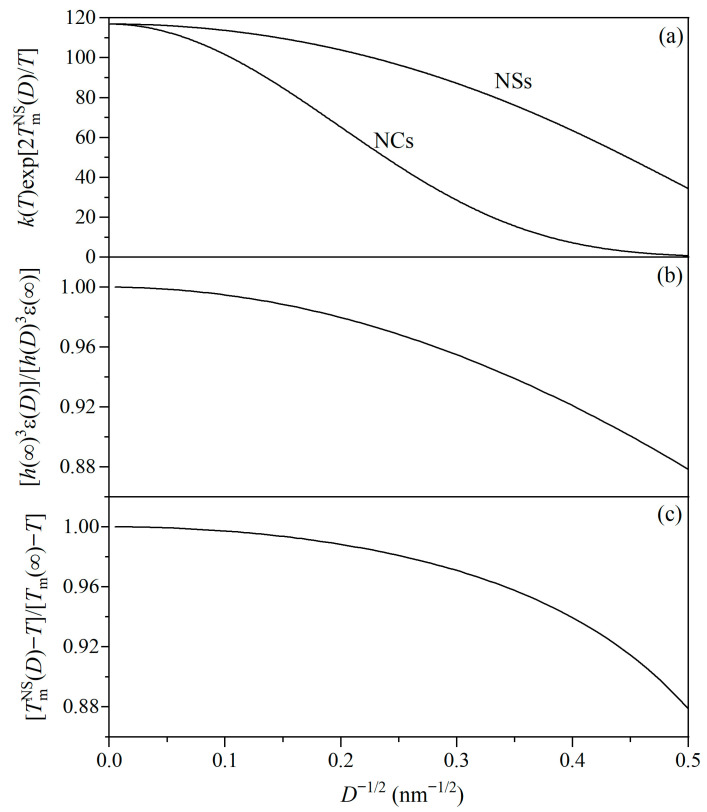
(**a**) *k*(*T*)exp [2$T_{m}^{\mathrm{NS}}$(*D*)/*T*], (**b**) [*h*(∞)^3^ε(*D*)]/[*h*(*D*)^3^ε(∞)], and (**c**) [$T_{m}^{\mathrm{NS}}$(*D*) − *T*]/[*T*_m_(∞) − *T*] as functions of *D*^−1/2^ for u-NSs of Fe using Equations (5)–(9) with *T* = 300 K, where the case of free NCs with $T_{m}^{\mathrm{NC}}$(*D*) is also plotted in (**a**) for comparison. See Table 1 for the necessary parameters.

To further understand the roles of activation energy, lattice expansion and bond weakening, and thermally driven effects in the reduction in σ(*T*,*D*), Figure 2 gives the curves of *k*(*T*)exp [2$T_{m}^{\mathrm{NS}}$(*D*)/*T*], [*h*(∞)^3^ε(*D*)]/[*h*(*D*)^3^ε(∞)], and [$T_{m}^{\mathrm{NS}}$(*D*) − *T*]/[*T*_m_(∞) − *T*] as functions of *D*^−1/2^ for u-NSs of Fe, where the case of free NCs is also given in Figure 2a. From Figure 2a, the contribution of activation energy to the reduction in σ(*T*,*D*) increases on lowering *D* for u-NSs and free NCs. At temperatures far below *T*_m_(∞), GBs are hard and exist as barriers to the dislocation movement. However, GBs start to soften due to the $T_{m}^{\mathrm{NS}}$(*D*) suppression, and the activation energy of GBs migration decreases, resulting in the reduction in σ(*T*,*D*). Moreover, the variation in u-NSs is weaker than that of free NCs because γ_gb_/γ_sv_ < 1. Figure 2b shows that the effects of lattice expansion and bond weakening on the reduction in σ(*T*,*D*) increase with lowering *D*. The lattice expansion of u-NSs at small *D* values leads to *Y*(*T*,*D*) suppression at room temperature [29]. As there is a proportional relationship between the shear modulus (*G*) and *Y*(*T*,*D*), *G*(*T*,*D*) also decreases with lowering *D*. Moreover, *h*(*D*) increases with lowering *D*, and thus the lattice resistance decreases accordingly, leading to easier dislocation movement and plastic deformation [55]. Moreover, the single bond energy weakening of u-NSs brings about a decline of local energy density. Due to the proportional relationship between *Y*(*T*,*D*) and the bond energy density [33], *Y*(*T*,*D*) and σ(*T*,*D*) also decrease. The thermally driven effect on the reduction in σ(*T*,*D*) is found to become more obvious with lowering *D* from Figure 2c. As *D* decreases, the ratio of low-coordination atoms at GBs to total atoms increases, resulting in the decrement of $T_{m}^{\mathrm{NS}}$(*D*). This means that u-NSs have high-temperature properties with lowering *D*, leading to the further reduction in σ(*T*,*D*).

As regards the strength of contributions from different effects, the contribution of activation energy to the reduction in σ(*T*,*D*) is up to 70% at *D*^−1/2^ = 0.5 nm^−1/2^ from Figure 2a, and the combined contribution of lattice expansion and bond weakening and the thermally driven effect increases up to around 24% at *D*^−1/2^ = 0.5 nm^−1/2^ from Figure 2b,c. This infers that the effects of lattice expansion and bond weakening and the thermally driven effect are relatively weak but cannot be neglected.

**Figure 3:**
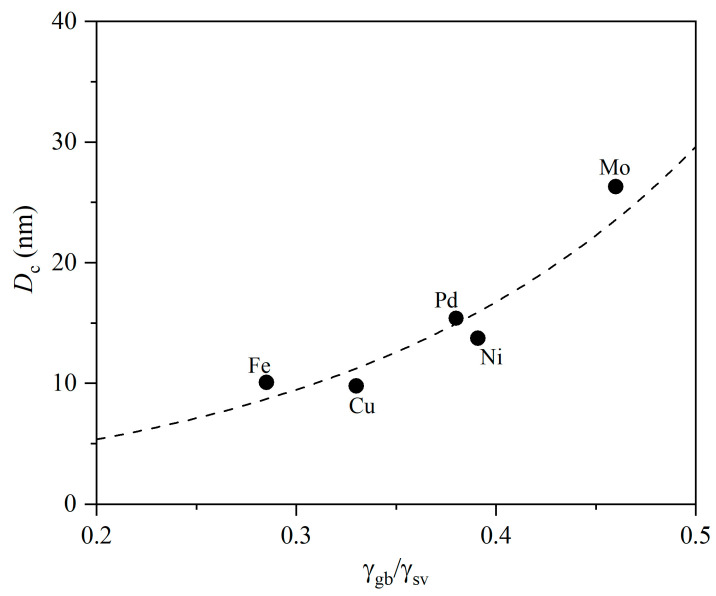
*D*_c_ as a function of γ_gb_/γ_sv_ for u-NSs of Fe, Cu, Ni, Pd, and Mo with *T* = 300 K. The dashed line is the exponential fit to the data. See Table 1 for the necessary parameters.

Figure 3 exhibits the curve of *D*_c_ as a function of γ_gb_/γ_sv_ for u-NSs of Fe, Cu, Ni, Pd, and Mo to reveal the role of γ_gb_. The plot shows that *D*_c_ increases significantly with increasing γ_gb_/γ_sv_ and *D*_c_ is about 10–30 nm, which is consistent with experimental results [27]. This suggests the vital and non-negligible role of γ_gb_ on *D*_c_ and σ(*T*,*D*).

**Figure 4:**
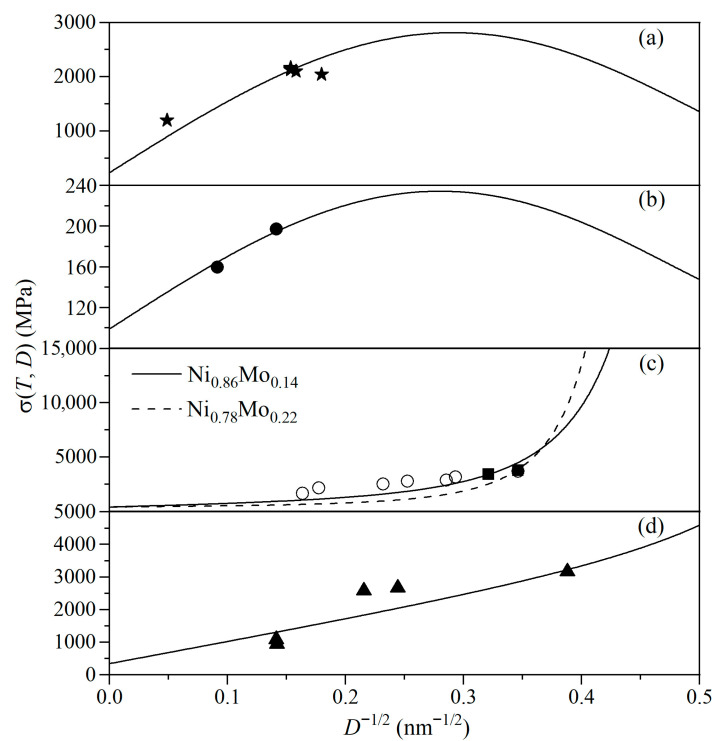
σ(*T*,*D*) as a function of *D*^−1/2^ for a-NSs of (**a**) Ni_0.56_Cu_0.44_, (**b**) Fe_99.985_Cu_0.015_, (**c**) Ni_0.86_Mo_0.14_ and Ni_0.78_Mo_0.22_, and (**d**) Fe_0.99_Zr_0.01_ using Equation (10) with *T* = 300 K. Symbols are experimental results with ★ [56] in (**a**) for a-NSs of Ni_0.56_Cu_0.44_, ● [12] in (**b**) for a-NSs of Fe_99.985_Cu_0.015_, ■ [14] in (**c**) for a-NSs of Ni_0.86_Mo_0.14_ and Ni_0.78_Mo_0.22_, respectively, and ▲ [15] in (**d**) for a-NSs of Fe_0.99_Zr_0.01_. Ni alloyed with other Mo contents (Ni_0.81_Mo_0.19_, Ni_0.87_Mo_0.13_, Ni_0.91_Mo_0.09_, Ni_0.94_Mo_0.06_, Ni_0.97_Mo_0.03_, and Ni_0.99_Mo_0.01_) denoted by ○ [14] is also shown in (**c**) for reference. See Table 2 for the necessary parameters.

Different γ_gb_ values are relevant to different alloying metals for a-NSs, and one can thus speculate that the variation in σ(*T*,*D*) with lowering *D* will be complicated. Figure 4 shows the σ(*T*,*D*) curves for a-NSs of Ni-Cu, Fe-Cu, Ni-Mo, and Fe-Zr. For the a-NSs of Ni-Cu and Fe-Cu shown in Figure 4a,b, σ(*T*,*D*) increases with lowering *D* and begins to decrease when *D* < *D*_c_, suggesting that the σ(*T*,*D*) of low-*T*_m_ a-NSs also follows the IHPR. In this case, the weak bonds of alloying atoms segregated at GBs result in the relaxation of local pressure and positive γ_gb_ values. Similarly to the IHPR of u-NSs, therefore, GBs become easier to move with lowering *D*, leading to the reduction in σ(*T*,*D*). For the a-NSs of Ni-Mo and Fe-Zr shown in Figure 4c,d, interestingly, σ(*T*,*D*) increases monotonically with lowering *D*, demonstrating the elimination of IHPR for high-*T*_m_ a-NSs. In this case, the segregation of alloying atoms also occurs at GBs. However, γ_gb_ becomes negative, and $T_{m}^{\mathrm{NS}}$(*D*) is increased, leading to the interface stiffening and thus an increase in the activation energy of boundary diffusion. Similarly to the dislocation pile-ups, this will also hinder the GBs’ movement, and subsequently σ(*T*,*D*) increases continuously with lowering *D* even below 10 nm, eliminating the IHPR observed for u-NSs and low-*T*_m_ a-NSs. Within the experimentally accessible *D* ranges, our model predictions show reasonable consistency with observed trends.

**Table 2:** Parameters of the selected alloys used in the model calculation.

	Ni_0.86_Mo_0.14_	Ni_0.78_Mo_0.22_	Fe_0.99_Zr_0.01_	Ni_0.56_Cu_0.44_	Fe_0.985_Cu_0.015_
*h*(∞) (nm) ^a^	0.309	0.316	0.313	0.294	0.312
*H*_m_(∞) (kJ/g·atom) ^a^	19.5	20.7	13.9	15.5	13.8
*T*_m_(∞) (K) ^a^	1889.8	1983.4	1812.2	1564.1	1802.2
*H*_b_(∞) (kJ/g-atom) ^a^	409.1	426.8	349.3	343.7	346.3
*T*_b_(∞) (K) ^a^	3429.6	3568.8	3149.5	3192.2	3135.0
γ_gb_(∞) (J/m^2^)	−2.46	−4.92	−0.15	0.77	0.93
γ_sv_(∞) (J/m^2^) ^b^	2.38	2.38	2.42	2.12	2.42
σ(300, ∞) (MPa) ^a^	386.8	381.8	344.9	232.5	98.9
*k*(*T*) (nm^1/2^) ^c^	2.71 × 10^−5^	4.96 × 10^−6^	1.11 × 10^−4^	1.75 × 10^−3^	4.59 × 10^−5^

^a^ For a-NSs (A*_x_*B_1−*x*_), *P* = *xP*_A_ + (1 − *x*)*P*_B_ as the first-order approximation, with *P* = *h*(∞), *H*_m_(∞), *T*_m_(∞), *H*_b_(∞), *T*_b_(∞), or σ(300, ∞). Our qualitative predictions (e.g., the presence or elimination of IHPR and the sign change in *γ*_gb_) are independent on reasonable variations in these alloy parameters. If we can obtain the corresponding accurate values, predictions will agree better with experimental results. For Zr, *h*(∞) = 0.310 nm, *H*_m_(∞) = 21 kJ/g·atom, *T*_m_(∞) = 2128 K, *H*_b_(∞) = 580 kJ/g·atom, *T*_b_(∞) = 4682 K [52], and σ(300, ∞) = 500 MPa [57]. ^b^ For Ni_0_._86_Mo_0_._14_, Ni_0_._78_Mo_0_._22_, Fe_0_._99_Zr_0_._01_, and Fe_0_._985_Cu_0_._015_, the γ_sv_(∞) values of the main elements (Ni and Fe) were used, while 0.56γ_sv-Fe_ + 0.44γ_sv-Cu_ was used for Ni_0_._56_Cu_0_._44_. ^c^ *k*(*T*) is obtained by fitting all experimental results listed in the caption of Figure 4 with the least-squares method in terms of Equation (10), where *T* = 300 K.

The present validation of Equation (10) is primarily based on room temperature datasets for simple metals and binary alloys. Application to chemically complex alloys, high-entropy systems, or thermally stabilized nanocrystalline materials will require extended parameterization that explicitly accounts for segregation thermodynamics and interface phase behavior.

## 4. Conclusions

In summary, based on the interface migration of atoms and *D*-dependent $T_{m}^{\mathrm{NS}}$(*D*), a unified thermodynamic formula was developed for distinct HPR and IHPR of u-NSs, low-*T*_m_ a-NSs, and high-*T*_m_ a-NSs. This formula predicts that the σ(*T*,*D*) of u-NSs increases with lowering *D* and begins to decrease when *D* < *D*_c_, following the IHPR. The reduction in σ(*T*,*D*) below *D*_c_ is attributed to the combining effect of activation energy, the thermally driven effect, and lattice expansion and bond weakening. For low-*T*_m_ a-NSs, σ(*T*,*D*) also follows the IHPR. However, the IHPR is eliminated for high-*T*_m_ a-NSs, where σ(*T*,*D*) monotonically increases with lowering *D* due to the negative interface energy induced by the aggregation of high-*T*_m_ alloying atoms at GBs. To verify the predictions, σ(*T*,*D*) values were compared with available experimental results, and good agreement can be found.

## Data Availability

The original contributions presented in this study are included in the article. Further inquiries can be directed to the corresponding author.

## References

[1] Konstantatos G., Sargent E.H. (2010). Nanostructured materials for photon detection. Nat. Nanotechnol..

[2] Kuchibhatla S.V., Karakoti A., Bera D., Seal S. (2007). One dimensional nanostructured materials. Prog. Mater Sci..

[3] Guo Y.G., Hu J.S., Wan L.J. (2008). Nanostructured materials for electrochemical energy conversion and storage devices. Adv. Mater..

[4] Gertsman V.Y., Birringer R., Gleiter H., Valiev R.Z. (1994). On the structure and strength of ultrafine-grained copper produced by severe plastic deformation. Scr. Metall. Mater..

[5] Hall E.O. (1951). The Deformation and Ageing of Mild Steel: III Discussion of Results. Proc. Phys. Soc. London Sect. B.

[6] Petch N.J. (1953). The Cleavage Strength of Polycrystals. J. Iron Steel Inst..

[7] Carlton C., Ferreira P. (2007). What is behind the inverse Hall–Petch effect in nanocrystalline materials?. Acta Mater..

[8] Ono N., Nowak R., Miura S. (2004). Effect of deformation temperature on Hall–Petch relationship registered for polycrystalline magnesium. Mater. Lett..

[9] Qin X., Zhu X., Gao S., Chi L., Lee J. (2002). Compression behaviour of bulk nanocrystalline Ni-Fe. J. Phys. Condens. Matter.

[10] Cordero Z.C., Knight B.E., Schuh C.A. (2016). Six decades of the Hall–Petch effect–a survey of grain-size strengthening studies on pure metals. Int. Mater. Rev..

[11] Naik S.N., Walley S.M. (2020). The Hall–Petch and inverse Hall–Petch relations and the hardness of nanocrystalline metals. J. Mater. Sci..

[12] Maruyama N., Sugiyama M., Hara T., Tamehiro H. (1999). Precipitation and Phase Transformation of Copper Particles in Low Alloy Ferritic and Martensitic Steels. Mater. Trans..

[13] Shen T., Schwarz R., Feng S., Swadener J., Huang J., Tang M., Zhang J., Vogel S., Zhao Y. (2007). Effect of solute segregation on the strength of nanocrystalline alloys: Inverse Hall–Petch relation. Acta Mater..

[14] Hu J., Shi Y.N., Sauvage X., Sha G., Lu K. (2017). Grain boundary stability governs hardening and softening in extremely fine nanograined metals. Science.

[15] Darling K.A., VanLeeuwen B.K., Koch C.C., Scattergood R.O. (2010). Thermal stability of nanocrystalline Fe–Zr alloys. Mater. Sci. Eng. A.

[16] Scattergood R.O., Koch C.C. (1992). A modified model for hall-petch behavior in nanocrystalline materials. Scr. Metall. Mater..

[17] Masumura R.A., Hazzledine P.M., Pande C.S. (1998). Yield stress of fine grained materials. Acta Mater..

[18] Van Swygenhoven H., Spaczer M., Caro A. (1999). Microscopic description of plasticity in computer generated metallic nanophase samples: A comparison between Cu and Ni. Acta Mater..

[19] Konstantinidis D.A., Aifantis E.C. (1998). On the “Anomalous” hardness of nanocrystalline materials. Nanostruct. Mater..

[20] Sun C.Q., Li S., Li C.M. (2005). Impact of Bond Order Loss on Surface and Nanosolid Mechanics. J. Phys. Chem. B.

[21] Jiang Q., Shi H., Zhao M. (1999). Melting thermodynamics of organic nanocrystals. J. Chem. Phys..

[22] Jiang Q., Zhang S., Zhao M. (2003). Size-dependent melting point of noble metals. Mater. Chem. Phys..

[23] Sherby O.D., Wadsworth J. (1989). Superplasticity-Recent advances and future directions. Prog. Mater. Sci..

[24] Herzig C., Mishin Y., Heitjans P., Kärger J. (2005). Grain boundary diffusion in metals. Diffusion in Condensed Matter.

[25] Chokshi A.H., Rosen A., Karch J., Gleiter H. (1989). On the validity of the Hall-Petch relationship in nanocrystalline materials. Scr. Metall..

[26] Meyers M.A., Mishra A., Benson D.J. (2006). Mechanical properties of nanocrystalline materials. Prog. Mater. Sci..

[27] Zhao M., Li J.C., Jiang Q. (2003). Hall–Petch relationship in nanometer size range. J. Alloy. Compd..

[28] Zhu Y.F., Lian J.S., Jiang Q. (2009). Modeling of the Melting Point, Debye Temperature, Thermal Expansion Coefficient, and the Specific Heat of Nanostructured Materials. J. Phys. Chem. C.

[29] Zhu Y.F., Zheng W.T., Jiang Q. (2011). Distinct Young’s modulus of nanostructured materials in comparison with nanocrystals. Phys. Chem. Chem. Phys..

[30] Guisbiers G., Herth E., Buchaillot L., Pardoen T. (2010). Fracture toughness, hardness, and Young’s modulus of tantalum nanocrystalline films. Appl. Phys. Lett..

[31] Schiøtz J., Tolla F.D.D., Jacobsen K.W. (1998). Softening of nanocrystalline metals at very small grain sizes. Nature.

[32] Zhu Y., Qin Q., Xu F., Fan F., Ding Y., Zhang T., Wiley B.J., Wang Z.L. (2012). Size effects on elasticity, yielding, and fracture of silver nanowires: In situ experiments. Phys. Rev. B.

[33] Liu X.J., Li J.W., Zhou Z.F., Yang L.W., Ma Z.S., Xie G.F., Pan Y., Sun C.Q. (2009). Size-induced elastic stiffening of ZnO nanostructures: Skin-depth energy pinning. Appl. Phys. Lett..

[34] Duscher G., Chisholm M.F., Alber U., Rühle M. (2004). Bismuth-induced embrittlement of copper grain boundaries. Nat. Mater..

[35] Zhu Y.F., Zheng W.T., Jiang Q. (2009). Modeling lattice expansion and cohesive energy of nanostructured materials. Appl. Phys. Lett..

[36] Ao Z.M., Li S., Jiang Q. (2008). The determination of Young’s modulus in noble metal nanowires. Appl. Phys. Lett..

[37] Jiang Q., Lu H.M. (2008). Size dependent interface energy and its applications. Surf. Sci. Rep..

[38] Chattopadhyay K., Goswami R. (1997). Melting and superheating of metals and alloys. Prog. Mater. Sci..

[39] Shao M., Peles A., Shoemaker K. (2011). Electrocatalysis on Platinum Nanoparticles: Particle Size Effect on Oxygen Reduction Reaction Activity. Nano Lett..

[40] Li H., Zhao M., Jiang Q. (2009). Cohesive Energy of Clusters Referenced by Wulff Construction. J. Phys. Chem. C.

[41] Tritsaris G.A., Greeley J., Rossmeisl J., Nørskov J.K. (2011). Atomic-Scale Modeling of Particle Size Effects for the Oxygen Reduction Reaction on Pt. Catal. Lett..

[42] Baletto F., Ferrando R. (2005). Structural properties of nanoclusters: Energetic, thermodynamic, and kinetic effects. Rev. Mod. Phys..

[43] Lu K., Lück R., Predel B. (1993). The interfacial excess energy in nanocrystalline Ni-P materials with different grain sizes. Scr. Metall. Mater..

[44] Fu H.H., Benson D.J., Meyers M.A. (2001). Analytical and computational description of effect of grain size on yield stress of metals. Acta Mater..

[45] Wu D., Zhang J., Huang J.C., Bei H., Nieh T.G. (2013). Grain-boundary strengthening in nanocrystalline chromium and the Hall–Petch coefficient of body-centered cubic metals. Scr. Mater..

[46] Sanders P.G., Eastman J.A., Weertman J.R. (1997). Elastic and tensile behavior of nanocrystalline copper and palladium. Acta Mater..

[47] Hughes G.D., Smith S.D., Pande C.S., Johnson H.R., Armstrong R.W. (1986). Hall-Petch strengthening for the microhardness of twelve nanometer grain diameter electrodeposited nickel. Scr. Metall..

[48] El-Sherik A.M., Erb U., Palumbo G., Aust K.T. (1992). Deviations from Hall-Petch behaviour in as-prepared nanocrystalline nickel. Scr. Metall. Mater..

[49] Schuh C.A., Nieh T.G., Yamasaki T. (2002). Hall–Petch breakdown manifested in abrasive wear resistance of nanocrystalline nickel. Scr. Mater..

[50] Nieman G.W., Weertman J.R., Siegel R.W. (1989). Microhardness of nanocrystalline palladium and copper produced by inert-gas condensation. Scr. Metall. Mater..

[51] Mohammadabadi A.S., Dehghani K. (2008). A new model for inverse Hall-Petch relation of nanocrystalline materials. J. Mater. Eng. Perform..

[52] The periodic table of the elements. https://www.webelements.com/.

[53] Tyson W., Miller W. (1977). Surface free energies of solid metals: Estimation from liquid surface tension measurements. Surf. Sci..

[54] Yoder K.B., Elmustafa A.A., Lin J.C., Hoffman R.A., Stone D.S. (2003). Activation analysis of deformation in evaporated molybdenum thin films. J. Phys. D Appl. Phys..

[55] Ohsawa K., Koizumi H., Kirchner H.O.K., Suzuki T. (1994). The critical stress in a discrete Peierls–Nabarro model. Philos. Mag. A.

[56] Pellicer E., Varea A., Sivaraman K.M., Pané S., Suriñach S., Baró M.D., Nogués J., Nelson B.J., Sort J. (2011). Grain Boundary Segregation and Interdiffusion Effects in Nickel–Copper Alloys: An Effective Means to Improve the Thermal Stability of Nanocrystalline Nickel. ACS Appl. Mater. Inter..

[57] Cai S., Daymond M., Khan A., Holt R., Oliver E. (2009). Elastic and plastic properties of β-Zr at room temperature. J. Nucl. Mater..

